# Identification of *N*-Acetyldopamine Dimers from the Dung Beetle *Catharsius molossus* and Their COX-1 and COX-2 Inhibitory Activities

**DOI:** 10.3390/molecules200915589

**Published:** 2015-08-27

**Authors:** Juan Lu, Qin Sun, Zheng-Chao Tu, Qing Lv, Pi-Xian Shui, Yong-Xian Cheng

**Affiliations:** 1 School of Medicine, Sichuan Medical University, 319 Zhongshan Road, Luzhou 646000, China; E-Mails: 18687525408@163.com (J.L.); sdy-0502@126.com (Q.S.); 2 State Key Laboratory of Phytochemistry and Plant Resources in West China, Kunming Institute of Botany, Chinese Academy of Sciences, 132 Lanhei Road, Kunming 650201, China; E-Mail: lvqing@mail.kib.ac.cn; 3 Guangzhou Institutes of Biomedicine and Health, Chinese Academy of Sciences, 190 Kaiyuan Road, Guangzhou 510530, China; E-Mail: tu_zhengchao@gibh.ac.cn

**Keywords:** *Catharsius molossus*, *N*-acetyldopamine dimers, COX-1, COX-2

## Abstract

Recent studies focusing on identifying the biological agents of *Catharsius molossus* have led to the identification of three new *N*-acetyldopamine dimers molossusamide A–C (**1−3**) and two known compounds **4** and **5**. The structures of the new compounds were identified by comprehensive spectroscopic evidences. Compound **4** was found to have inhibitory effects towards COX-1 and COX-2.

## 1. Introduction

**Figure 1:**
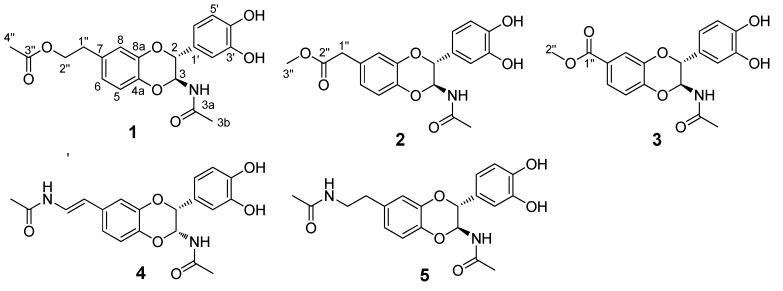
Structures of compounds (±)-**1**−(±)-**5***.*

*Catharsius molossus* (Linnaeus), one of the most widespread coprophagous species on the Earth, plays a vital role in ecosystems because it uses faeces as a source of food and nesting material. However, this insect is also important for its medicinal values. It was recorded in Shen-Nong-Ben-Cao-Jing, a classical ancient Chinese medical book, and has been used for centuries in China due to its multiple functions such as arresting convulsion, removing blood stasis, relaxing the bowels, and counteracting toxins [1]. Previous chemical investigations revealed that *C. molossus* contains melanin [2] and imidazole compounds [3]. The extract of *C. molossus* was found to have antitumor [4], anti-benign prostatic hyperplasia [5,6], and cardiovascular activities [7]. We have found that *C. molossus* extract possesses significant anxiolytic effects in mice [8]. Nonpeptide small molecules present in the insects often have intriguing chemical structures and biological activities, and some such compounds have been characterized from diverse insects [9], however, much still needs to be explored. As part of our broader efforts aimed at searching for and characterizing bioactive compounds extracted from insects, *C. molossus* was investigated. Three new *N*-acetyldopamine dimers **1**–**3** and two known compounds **4**–**5** were isolated (Figure 1). The biological activities of all the compounds were evaluated using cytotoxicity, MDCK cell based anti-influenza, EV71 inhibition and cyclooxygenase inhibitory assays. Below, we describe the isolation, structure identification, and biological evaluation of these compounds.

## 2. Results and Discussion

### Structural Identification

**Figure 2:**
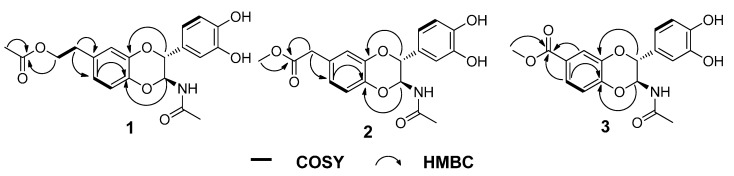
Key HMBC and COSY correlations of compounds **1**−**3**.

Compound **1** was obtained as a solid white substance. Its molecular formula was assigned as C_20_H_21_NO_7_, with eleven degrees of unsaturation, based on its HRESIMS data (*m*/*z* 410.1212 [M + Na]^+^, calcd for C_20_H_21_NO_7_Na, 410.1210). The ^1^H-NMR spectrum (Table 1) of **1** contains two typical ABX spin systems, [δ_H_ 6.82 (1H, d, *J* = 2.3 Hz, H-8), 6.75 (1H, dd, *J* = 8.5, 2.3 Hz, H-6), 6.81 (1H, d, *J* = 8.5 Hz, H-5), and 6.83 (1H, d, *J* = 1.7 Hz, H-2′), 6.73 (1H, dd, *J* = 8.5, 1.7 Hz, H-6′), 6.75 (1H, d, *J* = 8.5 Hz, H-5′)], suggesting the presence of two 1,3,4-trisubstituted benzene rings. The ^13^C-NMR and DEPT spectra (Table 1) contain resonances for 20 carbons, including two methyl, two aliphatic methylene (one oxygenated), eight methine (two oxygenated aliphatic, six olefinic), and eight quaternary carbons (two carbonyls, six olefinic including four oxygenated). The ^1^H-^1^H COSY spectrum (Figure 2) showed correlations between H-1″/H-2″, H-5/H-6, H-5′/H-6′, and H-2/H-3. The ^1^H- and ^13^C-NMR of **1** resemble those of **5**, differing in that an aliphatic methylene is present at C-2″ (δ_C_ 66.4). This assignment was confirmed by the observations of HMBC correlations (Figure 2) of H-2″ (δ_H_ 4.21)/C-3″ (δ_C_ 172.6), C-7 (δ_C_ 132.9) and H-4″ (δ_H_ 2.00)/C-3″ (δ_C_ 172.6). In addition, the presence of an ester carbonyl group in **1** instead of an amide in **5** are readily explained by the ^1^H- or ^13^C-NMR chemical shifts of H-2″ or C-2″. HMBC correlation between H-6 (dd, *J* = 8.5, 2.3 Hz)/C-4a suggested the side chain attached to C-7. The coupling constant for H-2 is 7.2 Hz, suggesting a *trans*-H-2/H-3 relationship. Compound **1** was isolated as a racemic mixture indicated by its optical rotation. Further chiral separation was not carried out in this study. Consequently, the structure of **1** was determined as shown in Figure 1 and named molossusamide A.

Compound **2** has a molecular formula C_19_H_19_NO_7_ (11 degrees of unsaturation) as derived from the HRESIMS data (*m*/*z* 396.1056 [M + Na]^+^, calcd for C_19_H_19_NO_7_Na, 396.1054). The ^1^H-NMR spectrum (Table 1) of **1** contains two typical ABX spin systems [δ_H_ 6.86 (1H, d, *J* = 1.8 Hz, H-8), 6.78 (1H, dd, *J* = 8.3, 1.8 Hz, H-6), 6.83 (1H, d, *J* = 8.3 Hz, H-5); 6.84 (1H, d, *J* = 1.6 Hz, H-2′), 6.74 (1H, dd, *J* = 8.3, 1.6 Hz, H-6′), 6.76 (1H, d, *J* = 8.3 Hz, H-5′)], suggesting the presence of two 1,3,4-trisubstituted benzene rings. The ^13^C-NMR and DEPT spectra contain resonances for 19 carbons including two methyl, one aliphatic methylene, eight methine (two oxygenated, six olefinic), and eight quaternary carbons (two carbonyls, six olefinic including four oxygenated). The above NMR data of **2** was quite similar to those of **1**. The main difference was a side chain at C-7. HMBC correlations (Figure 2) of H-1″/C-2″, C-6 and H-3″/C-2″ indicated the presence of C-1″–C-2″–O–C-3″, which was positioned at C-7 by the observation of HMBC correlation of H-6 (dd, *J* = 8.3, 1.8 Hz)/C-4a. The *trans*-form of H-2/H-3 was determined by the large coupling constant of H-2 (7.3 Hz). Of note, **2** was also isolated as a racemate, successive chiral separation was not carried out. As a result, the structure of **2** was determined as shown in Figure 1 and named molossusamide B.

Compound **3** possesses a molecular formula C_18_H_17_NO_7_ from its HRESIMS (*m*/*z* 360.1076 [M + H]^+^, calcd for C_18_H_18_NO_7_, 360.1078). The NMR data of **3** are extremely similar to those of **2**, differing in that an aliphatic methylene was missing in **3**, which was supported by HMBC correlations (Figure 2) of H-6, H-8, H-2″/C-1″. Similarly, the *trans*-configuration of H-2/H-3 was evident from the *J* value of H-2 (7.3 Hz). The racemic nature of **3** was indicated by its null optical rotation. Thus, the structure of **3** was determined as shown in Figure 1 and named molossusamide C.

The two known compounds were respectively identified as *cis*-2-(3′,4′-dihydroxyphenyl)-3-acetylamino-7-(*N*-acetyl-2″-amino-ethylene)-1,4-benzodioxane (**4**) [10] and *trans*-2-(3′,4′-dihydroxyphenyl)-3-acetyl-amino-7-(*N*-acetyl-2″-aminoethyl)-1,4-benzodioxane (**5**) [11], by comparison of their spectroscopic data with those in the literature. Both these compounds were isolated from this species for the first time. In addition, the ^1^H- and ^13^C-NMR data of **4** were assigned for the first time in this study (Table S1).

**Figure 3:**
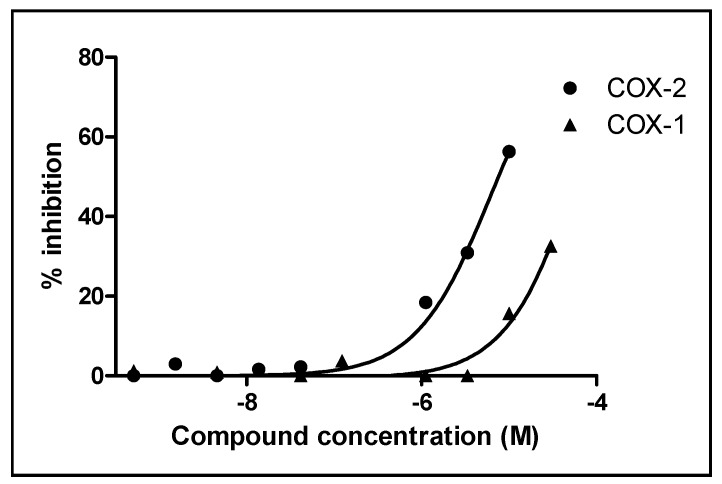
Inhibitory effects of compound **4** against COX-1 and COX-2.

Considering the traditional uses of *C. molossus* for the treatment of furunculosis, diarrhea [12] and cancer [13], the biological activities of racemic compounds **1**–**5** were evaluated using several assays including cytotoxicity in cancer cells, anti-virus (influenza and EV71) and anti-inflammation (COX-1/2). The results showed that only compound **4** exhibited inhibitory effects towards COX-1 and COX-2 with respective IC_50_ values of 78.85 μM and 6.43 μM (Figure 3 and Table S2) (celecoxib, with IC_50_ values of 54.55 μM and 0.015 μM for COX-1 and COX-2, respectively, was used as a positive control). All the compounds were inactive against cancer cells, influenza and EV71 (Table S3, Table S4 and Table S5). Because pyogenic infections are related to inflammation processes, the observations made in this study suggested that compound **4** might be responsible for the therapeutic applications of *C. molossus.*

**Table 1:** ^1^H- and ^13^C-NMR spectroscopic data of **1**−**3**.

Position	1		2		3	
	δ_H_ (*J* in Hz)	δ_C_, mult	δ_H_ (*J* in Hz)	δ_C_, mult	δ_H_ (*J* in Hz)	δ_C_, mult
2	4.68, d, 7.2	78.3, CH	4.70, d, 7.3	78.3, CH	4.74, d, 7.3	78.2, CH
3	5.66, d, 7.2	78.3, CH	5.68, d, 7.3	78.3, CH	5.78, d, 7.3	78.7, CH
5	6.81, d, 8.5	117.9, CH	6.83, d, 8.3	118.0, CH	6.96, d, 9.1	118.1, CH
6	6.75, dd, 8.5, 2.3	123.4, CH	6.78, dd, 8.3, 1.8	123.8, CH	6.84, dd, 9.1, 1.8	124.8, CH
7		132.9, qC		129.1, qC		128.2, qC
8	6.82, d, 2.3	118.3, CH	6.86, d, 1.8	118.8, CH	7.59, d, 2.0	119.5, CH
1′		128.7, qC		128.7, qC		124.8, qC
2′	6.83, d, 1.7	115.5, CH	6.84, d, 1.6	115.6, CH	7.58, d, 1.8	115.6, CH
3′		146.5, qC		146.5, qC		147.4, qC
4′		147.2, qC		147.2, qC		148.2, qC
5′	6.75, d, 8.5	116.1, CH	6.76, d, 8.3	116.1, CH	6.77, d, 8.3	116.2, CH
6′	6.73, dd, 8.5, 1.7	120.6, CH	6.74, dd, 8.3, 1.6	120.6, CH	6.75, dd, 8.3, 2.1	120.7, CH
1″	2.83, t, 6.9	35.3, CH_2_	3.56, s	40.9, CH_2_		168.1, qC
2″	4.21, t, 6.9	66.4, CH_2_		174.1, qC	3.85, s	52.5, CH_3_
3″		172.6, qC	2.04, s	52.5, CH_3_		
4″	2.00, s	20.8, CH_3_				
3a		173.3, qC		173.1, qC		173.3, qC
3b	1.87, s	22.6, CH_3_	1.87, s	22.6, CH_3_	1.87, s	22.6, CH_3_
4a		142.3, qC		142.8, qC		144.3, qC
8a		144.3, qC		144.3, qC		146.6, qC

## 3. Experimental Section

### 3.1. General Information

Optical rotations were recorded on a SEPA-300 polarimeter (Horiba, Kyoto, Japan). Column chromatography was performed on silica gel (200−300 mesh; Qingdao Marine Chemical, Inc., Qingdao, China), RP-18 (40−60 μm, Daiso Co., Osaka, Japan), MCI gel CHP 20P (75−150 μm, Mitsubishi Chemical Industries, Tokyo, Japan) and Sephadex LH-20 (Amersham Pharmacia, Uppsala, Biosciences, Sweden). UV spectra were recorded on a Shimadzu UV-2401PC spectrometer (Shimadzu, Kyoto, Japan). Semi-preparative HPLC was carried out using an Agilent 1200 liquid chromatograph (Agilent, Santa Clara, CA, USA), the column used was a 250 mm × 9.4 mm, i.d., 5 μm, Zorbax SB-C_18_. NMR spectra were recorded on a Bruker AM-400 or Avance III 600 or Avance III 800 NMR spectrometer (Bruker, Karlsruhe, Germany). Chemical shifts (δ) were expressed in ppm. EIMS and HREIMS were determined on an AutoSpec Premier P776 spectrometer (Waters, Milford, MA, USA). ESIMS and HRESIMS were measured on an API QSTAR Pulsar 1 spectrometer (Applied Biosystems/MDS Sciex, Foster City, CA, USA).

### 3.2. Insect Material

The whole bodies of *C. molossus* were purchased from Ju-Hua-Cun herbal market in Kunming of Yunnan Province, China, in September 2012. The material was identified by Prof. Z.-Y. Yan at Chengdu University of Traditional Chinese Medicine. A voucher specimen (CHYX0581) was preserved at the State Key Laboratory of Phytochemistry and Plant Resources in West China, Kunming Institute of Botany, Chinese Academy of Sciences, China.

### 3.3. Extraction and Isolation

The dried powder of *C. molossus* (50 kg) was soaked using 70% EtOH (3 × 360 L × 24 h) to give a crude extract, which was suspended in water and adjusted pH to 1–2 followed by extraction with EtOAc (3 × 10 L) to afford an EtOAc soluble extract (800 g). The EtOAc extract was divided into three parts (Fr.1−Fr.3) using silica gel solid phase extraction eluted with petroleum ether, CH_2_Cl_2_ and MeOH. Fr.3 (55 g, methanol soluble part) was separated by MCI gel CHP 20P eluted with gradient aqueous MeOH (20%–95%) to provide eleven portions (Fr.3.1−Fr.3.11). Fr.3.6 (24.4 g) was filtered via Sephadex LH-20 (MeOH) to produce three portions (Fr.3.6.1−Fr.3.6.3). Fr.3.6.1 (6 g) was submitted to RP-18 column chromatography (MeOH/H_2_O, 50%–75%) followed by preparative TLC (2 drops formic acid in CHCl_3_/MeOH, 14:1 vol/vol) and semi-preparative HPLC (MeOH/H_2_O, 50%) to yield **1** (4.1 mg, Rt = 17.1 min). Fr.3.6.2 (5 g) was fractionated on a RP-18 column (MeOH/H_2_O, 25%–55%) to give three portions (Fr.3.6.2.1–Fr.3.6.2.3). Among them, Fr.3.6.2.1 (50 mg) was purified by preparative TLC (CHCl_3_/MeOH, 9:1 vol/vol) and then semi-preparative HPLC (MeOH/H_2_O, 45%) to afford **2** (1.2 mg, Rt = 19.6 min) and **3** (1.6 mg, Rt = 20.2 min). Fr.3.6.2.3 (45.3 mg) was purified by preparative TLC (CHCl_3_/acetone, 2.5:1 vol/vol) to yield **4** (10 mg). Fr.3.5 (24 g) was separated by Sephadex LH-20 chromatography (MeOH) to provide six portions (Fr.3.5.1−Fr.3.5.6). Fr.3.5.4 (8 g) was applied to a RP-18 column (MeOH/H_2_O, 20%–50%), followed by semi-preparative HPLC (MeOH/H_2_O, 35%) to give **5** (10 mg, Rt = 11.7 min).

### 3.4. Compound Characterization

*(±)-Molossusamide A* (**1**). Yellowish solid; ${\left[\alpha \right]}_{\text{D}}^{26}$ −1.3 (*c* 0.26, MeOH); UV (MeOH) λ_max_ (log ε): 204 (4.88), 221 (4.34), 283 (3.93) nm; ^1^H- (CD_3_OD, 400 MHz) and ^13^C-NMR (CD_3_OD, 200 MHz) data, see Table 1; ESIMS: *m*/*z* 387 [M]^+^; HRESIMS: *m*/*z* 410.1212 [M + Na]^+^ (calcd for C_20_H_21_NO_7_Na, 410.1210).

*(±)-Molossusamide B* (**2**). Yellowish solid; ${\left[\alpha \right]}_{\text{D}}^{26}$ −7.8 (*c* 0.24, MeOH); UV (MeOH) λ_max_ (log ε): 204 (4.65), 223 (4.29), 254 (3.96), 278 (3.91), 319 (3.38) nm; ^1^H- (CD_3_OD, 400 MHz) and ^13^C-NMR (CD_3_OD, 150 MHz) data, see Table 1; ESIMS: *m*/*z* 396 [M + Na]^+^; HRESIMS: *m*/*z* 396.1056 [M + Na]^+^ (calcd for C_19_H_19_NO_7_Na, 396.1054).

*(±)-Molossusamide C* (**3**). Yellow solid; ${\left[\alpha \right]}_{\text{D}}^{26}$ +1.4 (*c* 0.33, MeOH); UV (MeOH) λ_max_ (log ε): 204 (4.54), 217 (4.40), 254 (4.04), 282 (3.91) nm; ^1^H- (CD_3_OD, 400 MHz) and ^13^C-NMR (CD_3_OD, 200 MHz) data, see Table 1; ESIMS: *m*/*z* 717 [2M − H]^−^; HRESIMS: *m*/*z* 360.1076 [M + H]^+^ (calcd for C_18_H_18_NO_7_, 360.1078).

### 3.5. Cytotoxicity Assay

Cell lines, K562, MCF-7, A549, Huh-7, Hela, DU145, H1975, and A431 were purchased from the Shanghai Cell Bank, Chinese Academy of Sciences (Shanghai, China). Cells were routinely grown and maintained in mediums RPMI or DMEM with 10% FBS and with 1% penicillin/streptomycin. All cell lines were incubated in a Thermo/Forma Scientific CO_2_ Water Jacketed Incubator (Greenbelt Maryland, MD, USA) with 5% CO_2_ in air at 37 °C. Cell viability was determined by the CCK8 (Dojindo, Kyushu, Japan) assay. Cells were seeded at a density of 400–800 cells/well in 384 well plates and treated with various concentrations of compounds or solvent control. After 72 h incubation, CCK8 reagent was added, and absorbance was measured at 450 nm using Envision 2104 multi-label Reader (Perkin Elmer, Waltham, MA, USA)*.*

### 3.6. MDCK Cell-Based Anti-Influenza Assay

Cell-based anti-influenza virus inhibitor screening was based on the principle of cytopathic effect (CPE) protection assay. Madin-Darby canine kidney (MDCK) cells cultured to approximately 90% confluence were detached with 0.25% Trypsin-EDTA (Invitrogen, Shanghai, China), washed and re-suspended in complete EMEM, 2.5 × 10^4^ MDCK cells were plated in triplicate in a 96-well plate and incubated overnight at 37 °C in a humidified 5% CO_2_ incubator. The confluent MDCK monolayers cells were rinsed twice with Hanks’ solution devoid of serum, and then the cells were treated with 50 μL medium with 1 mg/mL TPCK and 0.3% BSA and infected by different influenza virus strains at a multiplicity of infection (MOI) of 0.01 PFU/cell. After 2.0 h incubation, serially diluted compounds were added. After 3 day incubation, the medium was removed and 50 μL medium containing 5 μL CCK8 reagent was added into each well followed by additional 2 h incubation, the absorbance was measured at 450 nm using an UV-star-Microplates Synergy HT plate reader (BioTek, Winnooski, VT, USA).

### 3.7. EV71 Inhibition CPE Assay

EV71 (GZ-08-02 strain, GenBank Accession No. FJ360545) was originally isolated and identified by the Guangzhou Children’s Hospital, Guangzhou, China [14]. The CPE induced by EV71 infection was measured in Vero cells with CCK8 assay. Confluent VERO cells were plated into 384-well plate and inoculated in triplicate with the mixture of 100 times of the 50% tissue culture infectious dose (TCID_50_) of EV71, and serially diluted compounds in DMEM supplemented with 2% FCS. After that, the cells were incubated at 37 °C for additional 72 h. Then fresh medium containing CCK8 were added, and incubated in 37 °C for 2 h. the A450 was measured with an Envision Plate Reader (PerkinElmer).

### 3.8. Cyclooxygenase (COX) Inhibitory Assay

Compounds were evaluated for COX inhibitory activity *in vitro* by using Cayman’s COX Fluorescent Inhibitor Screening Assay Kit (Cayman Chemical Company, Ann Arbor, MI, USA) as previously described methods [15].

## 4. Conclusions

Three new and two known *N*-acetyldopamine dimers were isolated from dung beetle, and their structures were characterized by spectroscopic methods. One known compound was found to have inhibitory activities against COX-1 and COX-2. This contribution adds new facets to the chemistry and biological activity of insect-derived nonpeptide small molecules.

## Supplementary Materials

Supplementary materials can be accessed at: http://www.mdpi.com/1420-3049/20/09/15589/s1.

## References

[1] Wang X.B., Yang Y.H. (2002). Dung beetles use. J. Exter. Ther. TCM.

[2] Xin C., Ma J.H., Tan C.J., Yang Z., Ye F., Long C., Ye S., Hou D.B. (2015). Preparation of melanin from *Catharsius molossus* L. and preliminary study on its chemical structure. J. Biosci. Bioeng..

[3] Suenaga K., Shimogawa H., Nakagawa S., Uemura D. (2001). *Catharsitoxins* from the Chinese remedy qiung laug. Tetrahedron Lett..

[4] Chen Z.H., Guan Y.M., Ou S.P., Zhou W.Q., Yang Y. (2012). Medicinal dung beetle effective parts and pharmacological research progress. Chin. Tradit. Pat. Med..

[5] Zhao X.M., Zhu M., Yang M., Tao K., Wang J.X. (2006). The study of *Catharsius molossus* L. on experimental prostatic hyperplasia. Pharmacol. Clin. Chin. Mater. Med..

[6] Kang H.J., Zhang X., Hou Y.H., Wu Y., Xu L.S., Sun L. (2012). Protective effect of Radix *Gentiana Macrophylla* on acute liver injury induced by CCl_4_ in mice. Pharmacol. Clin. Chin. Mater. Med..

[7] Hou X.M., Zhang S.J., Jia Y.F., Bai J.L., Li J.H. (2014). Ancient and modern application research of *Catharsius molossus* L. J. Hebei TCM Pharmacol..

[8] Cheng Y.X., Chen X.L., Bu W. (2011). Manufacture of Anxiolytics Using *Catharsius molossus* Extract as Active Component and Application Thereof.

[9] Harborne J.B. (2001). Twenty-five years of chemical ecology. Nat. Prod. Rep..

[10] Xu M.Z., Lee W.S., Han J.M., Oh H.W., Park D.S., Tian G.R., Jeong T.S., Park H.Y. (2006). Antioxidant and anti-inflammatory activities of *N*-acetyldopamine dimers from *Periostracum cicadae*. Bioorg. Med. Chem..

[11] Noda N., Kubota S., Miyata Y., Miyahara K. (2000). Optically active *N*-acetyldopamine dimer of the crude drug “Zentai”, the cast-off shell of the cicada, *Cryptotympana* sp. Chem. Pharm. Bull..

[12] Hou X.M., Zhang S.J., Jia Y.F., Bai J.L., Li J.H. (2014). Ancient and present applications of *Catharsius molossus*. J. Hebei TCM Pharmacol..

[13] Yu Y., Sun G.Z. (2014). Experience of Professor Guizhi Sun in treating tumor using insects. China J. Tradit. Chin. Med. Pharm..

[14] Ding N.Z., Wang X.M., Sun S.W., Song Q., Li S.N., He C.Q. (2009). Appearance of mosaic enterovirus 71 in the 2008 outbreak of China. Virus Res..

[15] Tang J.J, Fang P., Xia H.L., Tu Z.C., Hou B.Y., Yan Y.Y., Di L., Zhang L., Cheng Y.X. (2015). Constituents from the edible Chinese black ants (*Polyrhachis dives*) showing protective effects on rat mesangial cells and anti-inflammatory activity. Food Res. Int..

