# Supplementary material for: Identification of N-Acetyldopamine Dimers from the Dung Beetle Catharsius molossus and Their COX-1 and COX-2 Inhibitory Activities
Source: Molecules. 2015 Aug 27;20(9):15589–96. doi: 10.3390/molecules200915589 (PMC6331819; doi:10.3390/molecules200915589)
Supplement: Supplementary file 1 [file molecules-20-15589-s001.pdf]

Contents of Supporting Information

Figure S1. ^{1}H-NMR spectrum of **1** in CD_3OD.

Figure S2. ^{13}C-NMR and DEPT spectra of **1** in CD_3OD.

Figure S3. HSQC spectrum of **1** in CD_3OD.

Figure S4. HMBC spectrum of **1** in CD_3OD.

Figure S5. ^{1}H-^{1}H COSY spectrum of **1** in CD_3OD.

Figure S6. HRESIMS spectrum of **1**.

Figure S7. ^{1}H-NMR spectrum of **2** in CD_3OD.

Figure S8. ^{13}C-NMR and DEPT spectra of **2** in CD_3OD.

Figure S9. HSQC spectrum of **2** in CD_3OD.

Figure S10. HMBC spectrum of **2** in CD_3OD.

Figure S11. ^{1}H-^{1}H COSY spectrum of **2** in CD_3OD.

Figure S12. HRESIMS spectrum of **2**.

Figure S13. ^{1}H-NMR spectrum of **3** in CD_3OD.

Figure S14. ^{13}C-NMR and DEPT spectra of **3** in CD_3OD.

Figure S15. HSQC spectrum of **3** in CD_3OD.

Figure S16. HMBC spectrum of **3** in CD_3OD.

Figure S17. ^{1}H-^{1}H COSY spectrum of **3** in CD_3OD.

Figure S18. HRESIMS spectrum of **3**.

Figure S19. ^{1}H-NMR spectrum of **4** in CD_3OD.

Figure S20. ^{13}C-NMR and DEPT spectra of **4** in CD_3OD.

Figure S21. HSQC spectrum of **4** in CD_3OD.

Figure S22. HMBC spectrum of **4** in CD_3OD.

Table S1. The ^{1}H- and ^{13}C-NMR spectroscopic data of **4**.

Table S2. COX-1 and COX-2 inhibitory activities of the compounds.

Table S3. Cytotoxicity of the compounds.

Table S4. MDCK cell-based anti-influenza activities of the compounds.

Table S5. EV71 inhibitory activities of the compounds.

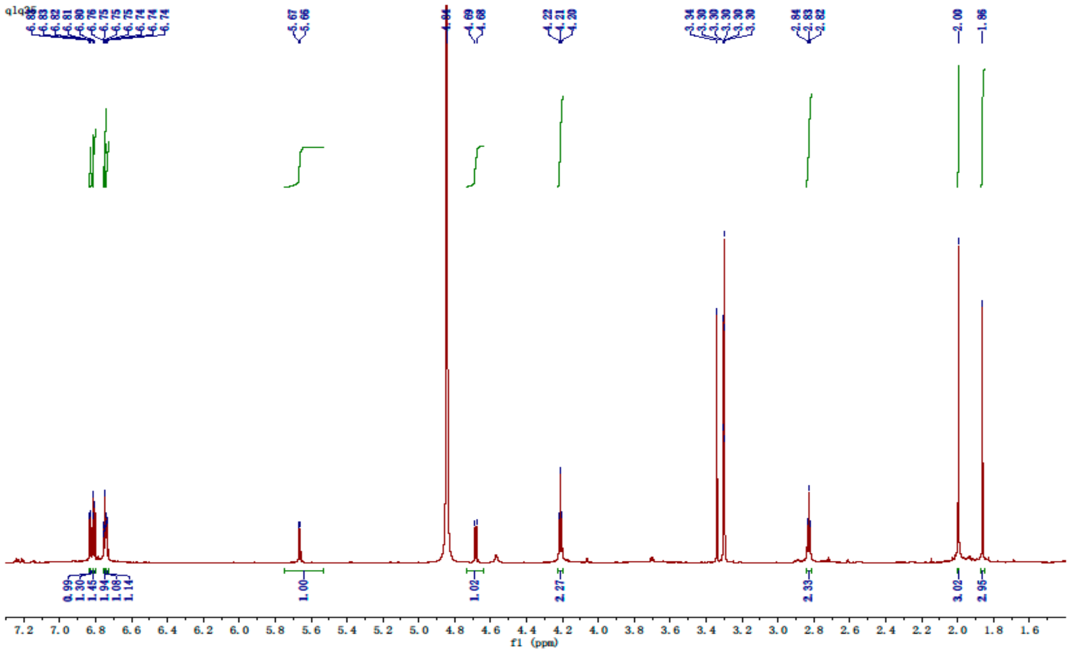

Figure S1. ^{1}H-NMR spectrum of **1** in CD_3OD.

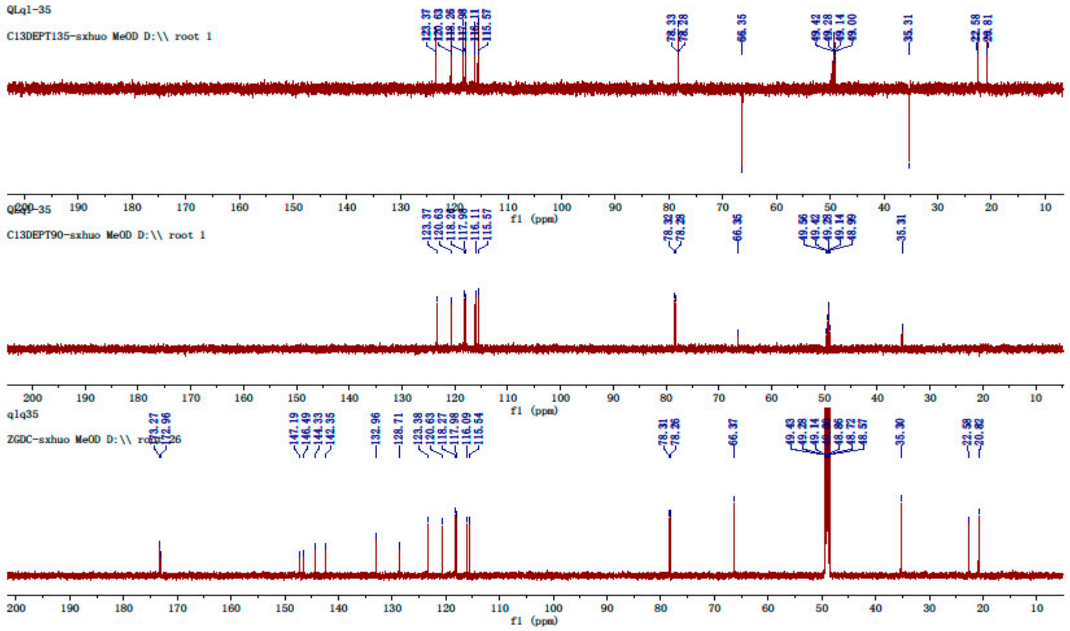

Figure S2. ^{13}C-NMR and DEPT spectra of **1** in CD_3OD.

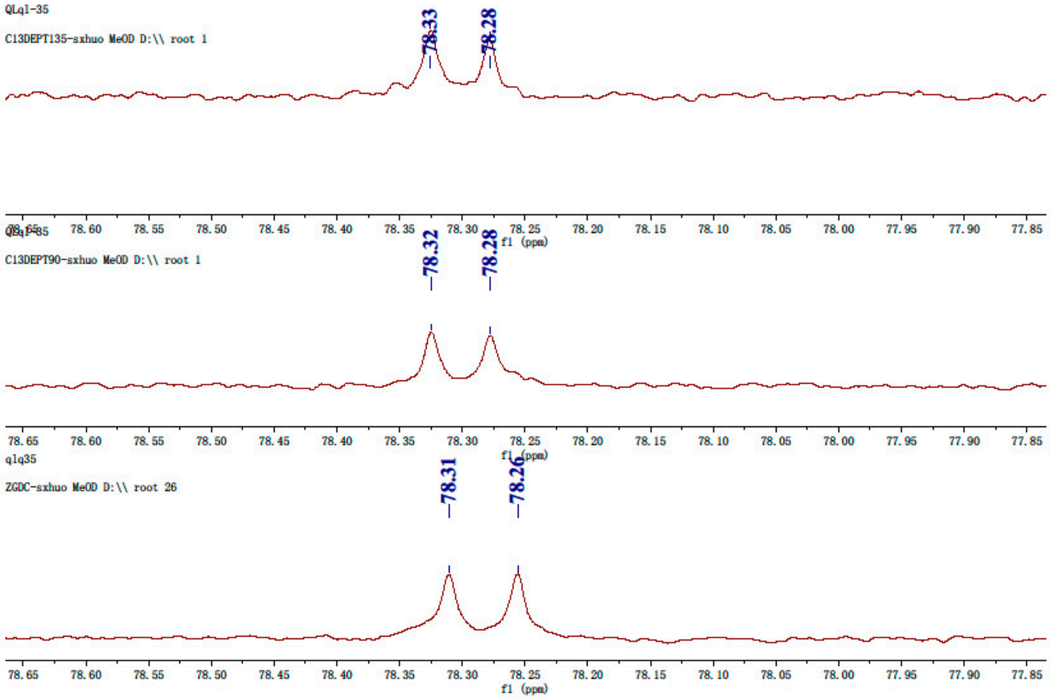

Enlarged ^{13}C-NMR and DEPT spectra of **1** (C-2,C-3) in CD_3OD.

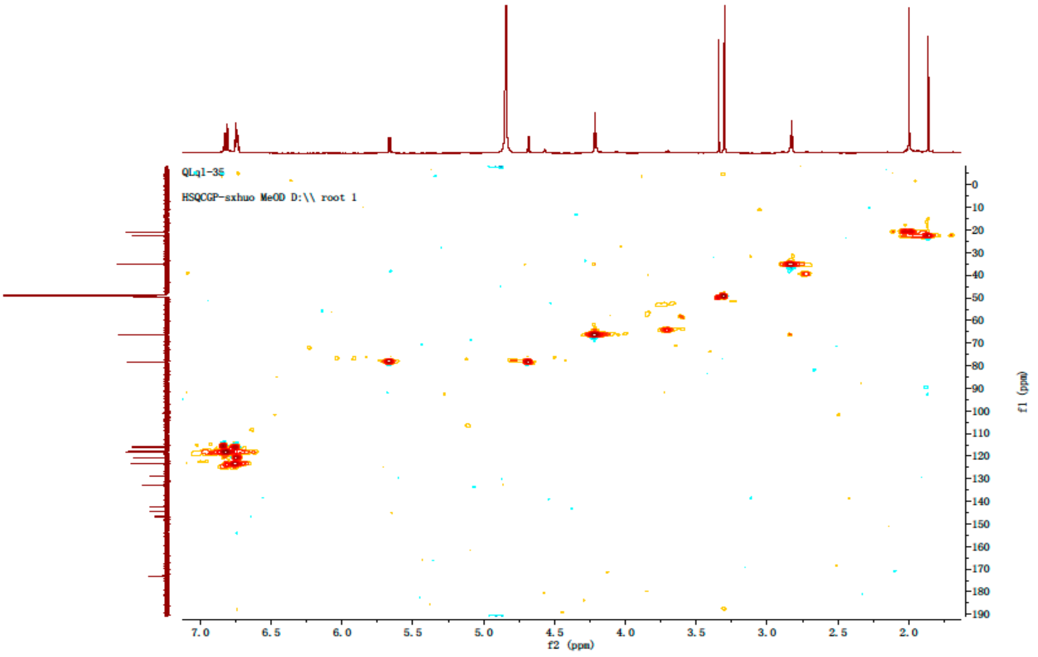

Figure S3. HSQC spectrum of **1** in CD_3OD.

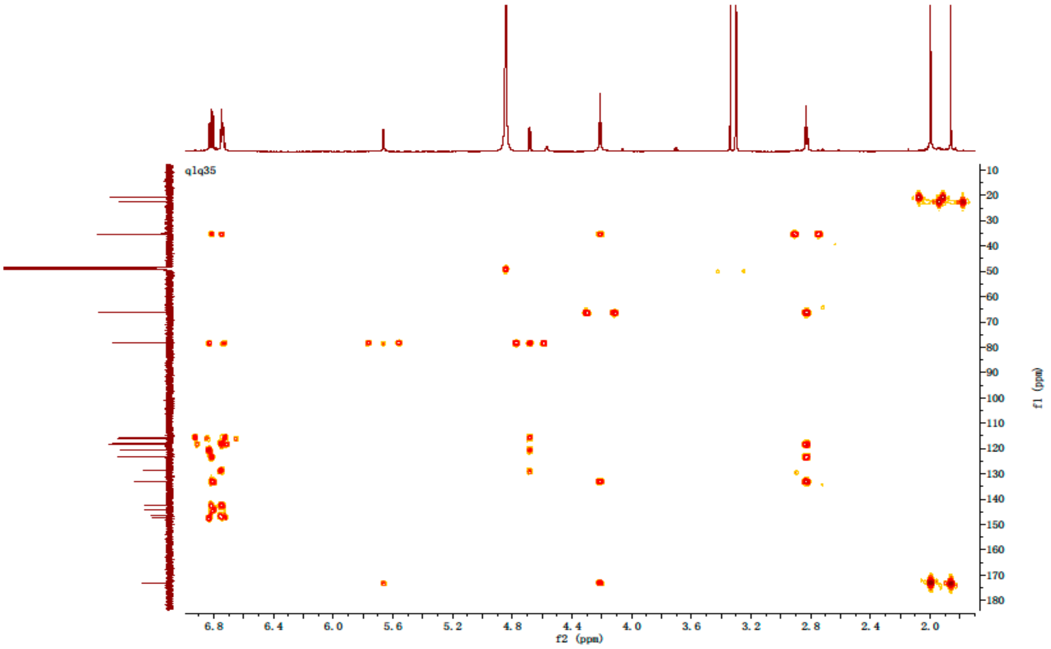

Figure S4. HMBC spectrum of **1** in CD_3OD.

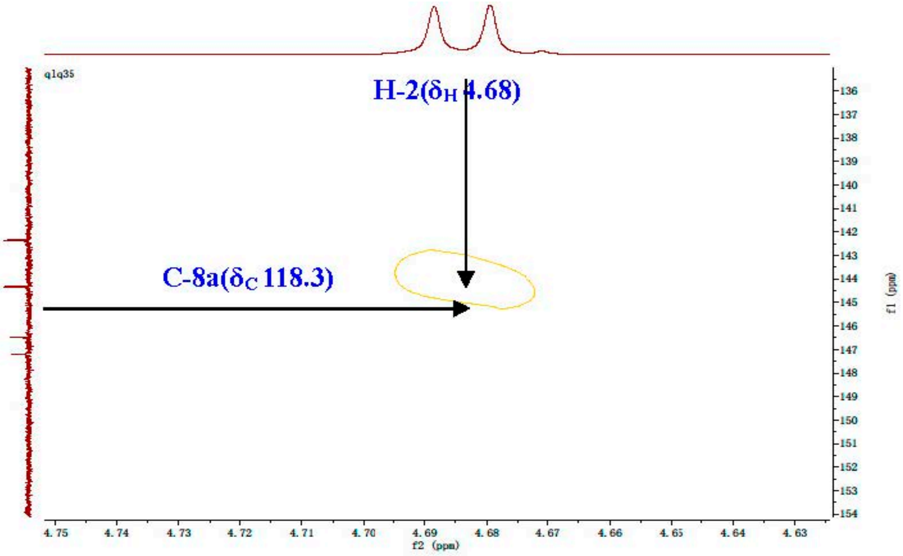

Enlarged HMBC spectrum of **1** (key correlations of H-2/C-8a) in CD_3OD.

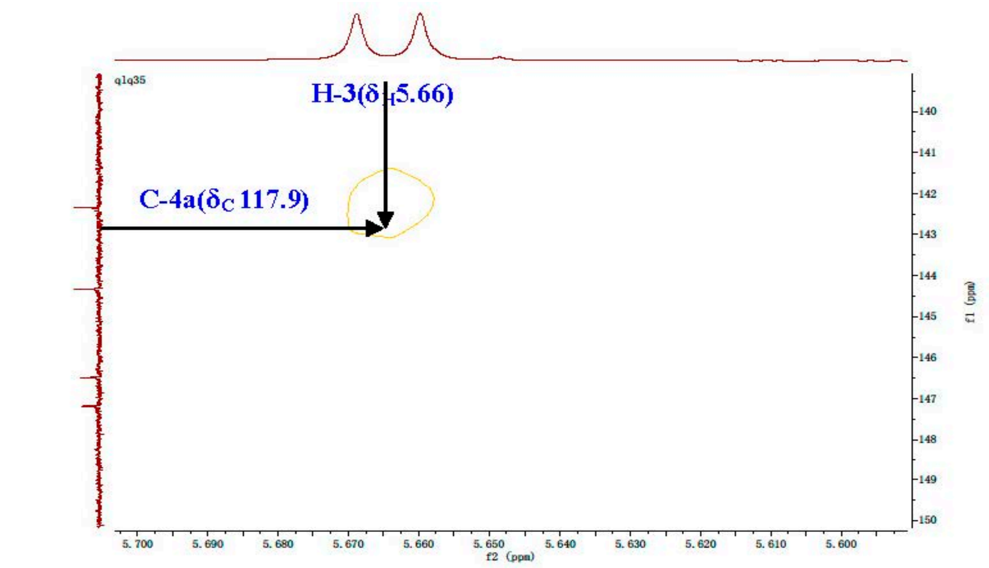

Enlarged HMBC spectrum of **1** (key correlations of H-3/C-4a) in CD_3OD.

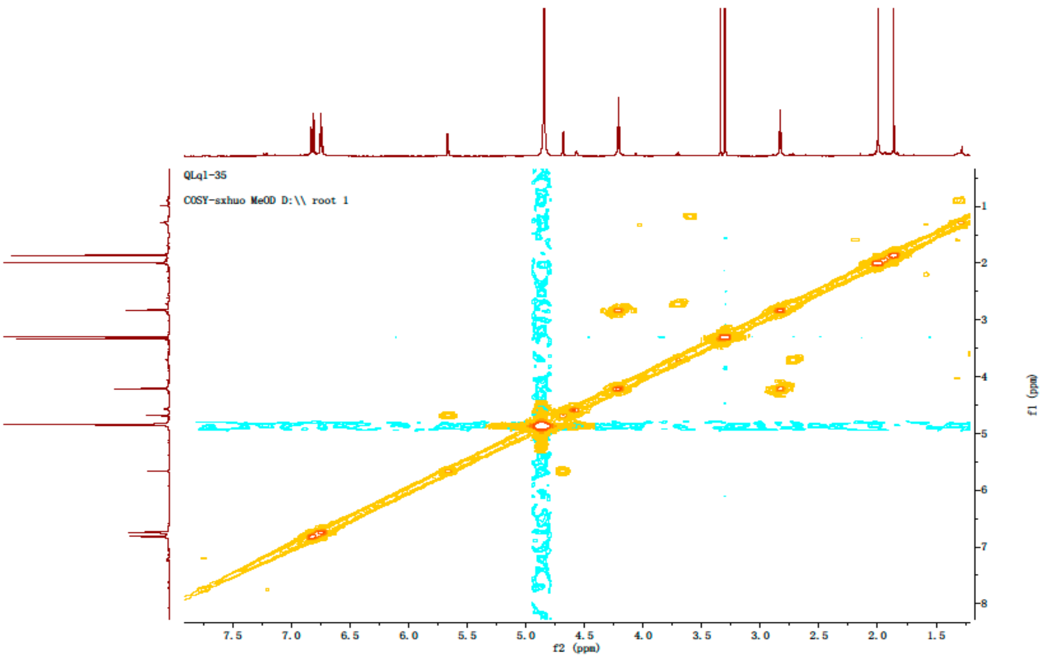

Figure S5. ^1H-^1H COSY spectrum of **1** in CD_3OD.

Qualitative Analysis Report

**Data Filename**	qlql-35.d	**Sample Name**	qlql-35
**Sample Type**	Sample	**Position**	P1-A1
**Instrument Name**	Instrument 1	**User Name**	
**Acq Method**	SIBU.m	**Acquired Time**	4/13/2015 2:06:28 PM
**IRM Calibration Status**	Success	**DA Method**	Default.m
**Comment**			

Sample Group **Info.**

Acquisition SW Version 6200 series TOF/6500 series Q-TOF B.05.01 (B5125.2)

User Spectra

Fragmentor Voltage	Collision Energy	Ionization Mode
135	0	ESI

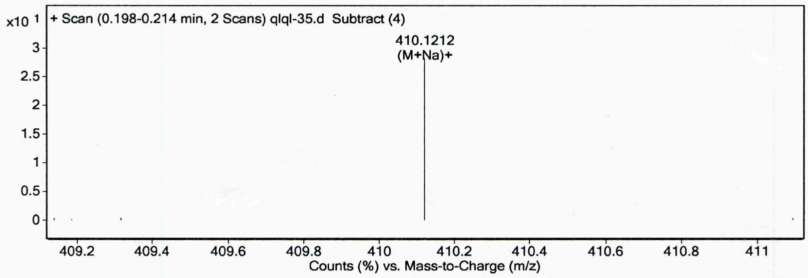

Peak List

m/z	z	Abund	Formula	Ion
274.2745	1	24310.29		
302.3055	1	10523.09		
318.3002	1	12763.09		
381.2981	1	9396.49		
388.1392	1	8674.9		
410.1212	1	15832.46	C20 H21 N O7	(M+Na)+

Formula Calculator Element Limits

Element	Min	Max
C	3	60
H	0	120
N	0	10
O	0	30

Formula Calculator Results

Formula	CalculatedMass	CalculatedMz	Mz	Diff. (mDa)	Diff. (ppm)	DBE
C20 H21 N O7	387.1318	410.1210	410.1212	-0.3	-0.9	11.0000

--- End Of Report ---

Figure S6. HRESIMS spectrum of **1**.

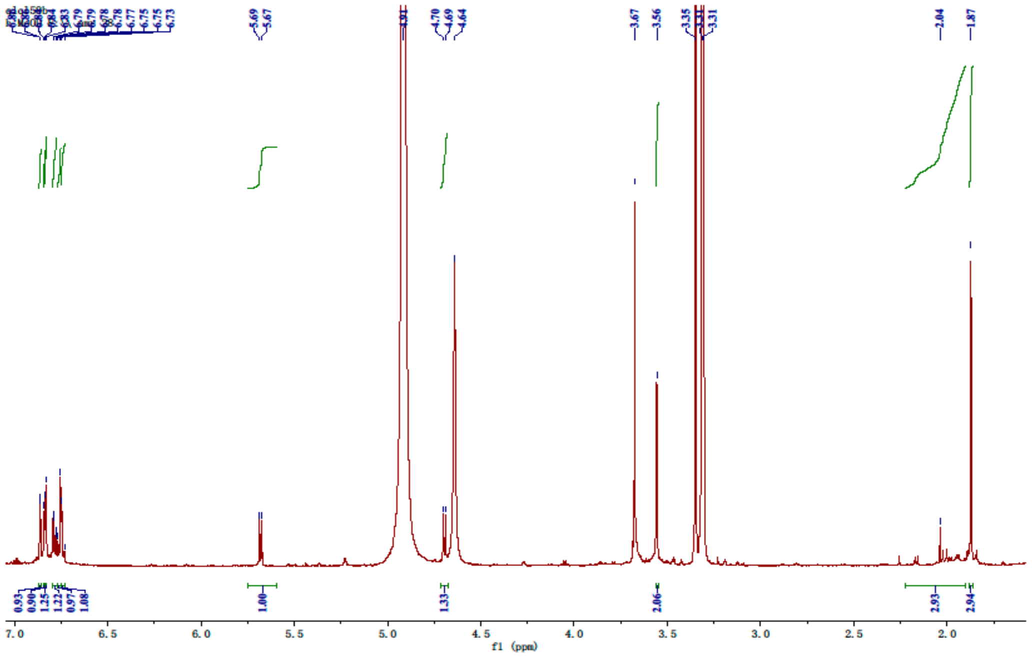

Figure S7. ^{1}H-NMR spectrum of **2** in CD_3OD.

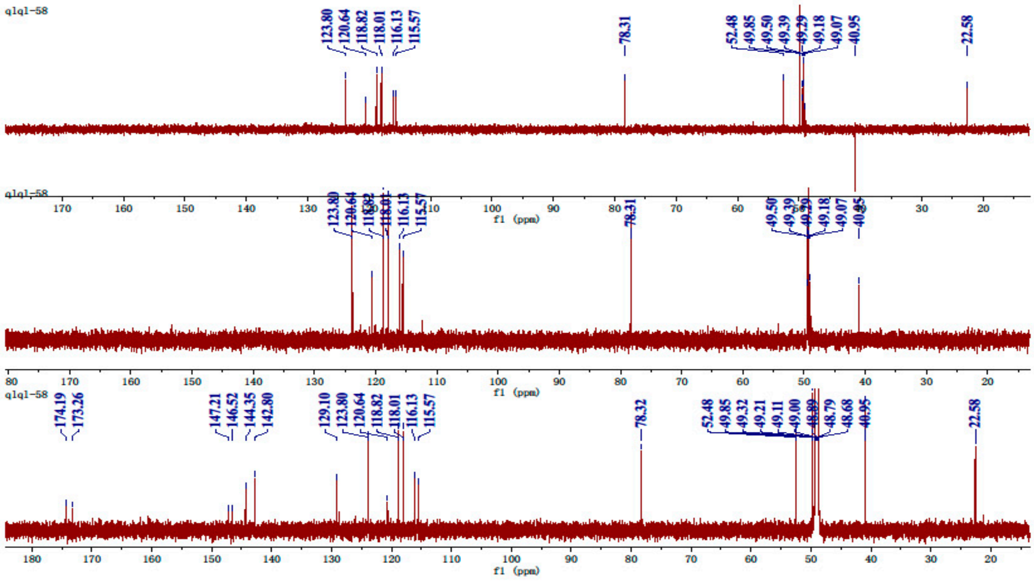

Figure S8. ^{13}C-NMR and DEPT spectra of **2** in CD_3OD.

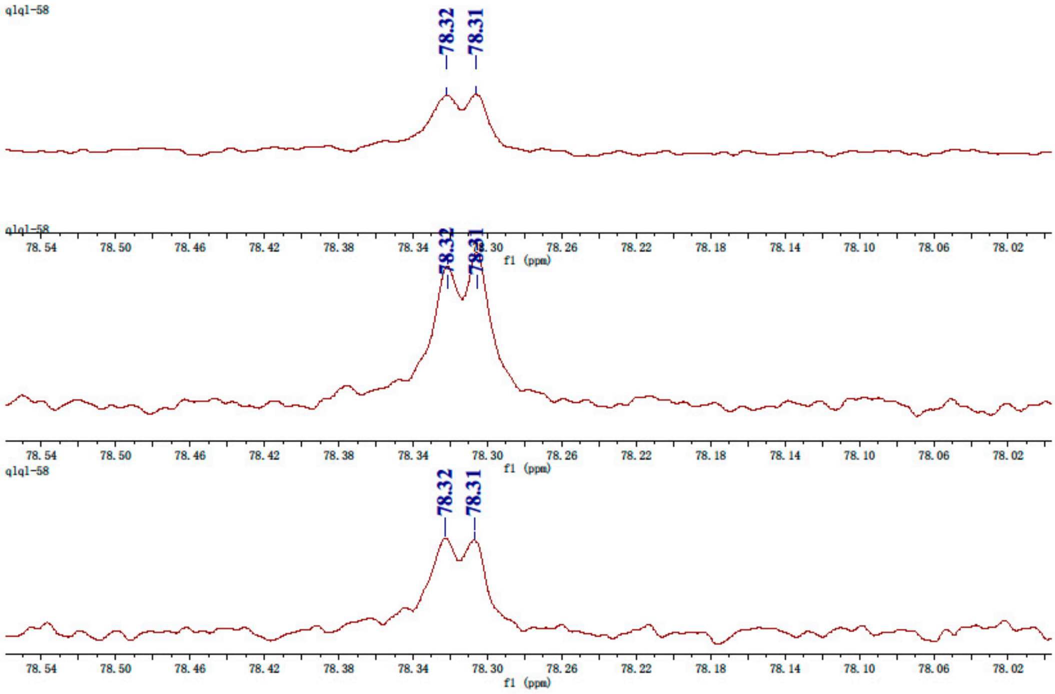

Enlarged ^{13}C-NMR and DEPT spectra of **2** (C-2, C-3) in CD_3OD.

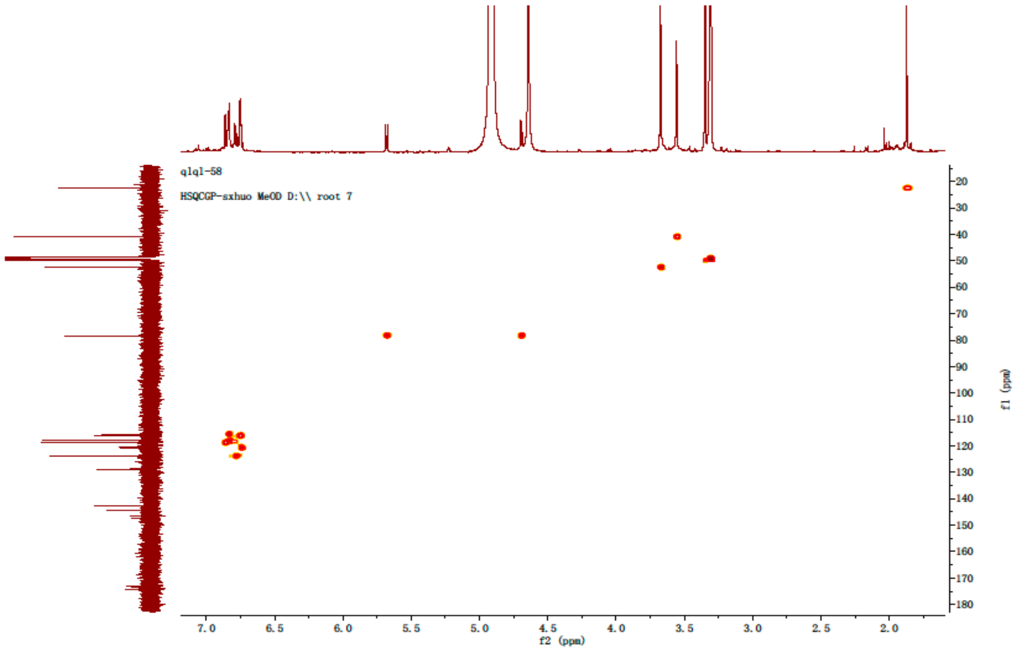

Figure S9. HSQC spectrum of **2** in CD_3OD.

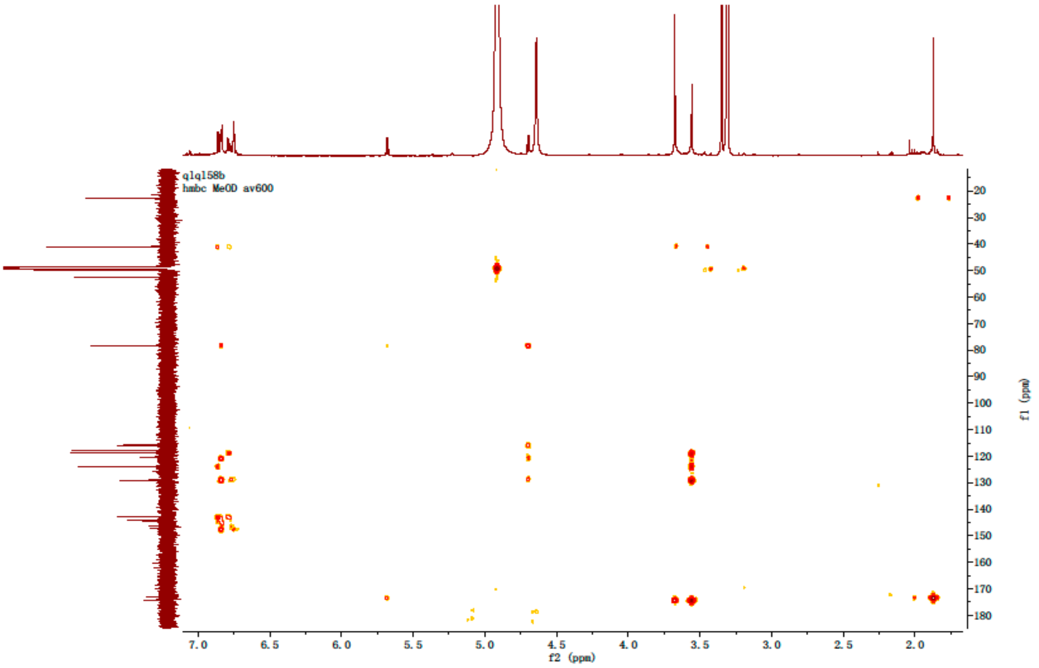

Figure S10. HMBC spectrum of **2** in CD_3OD.

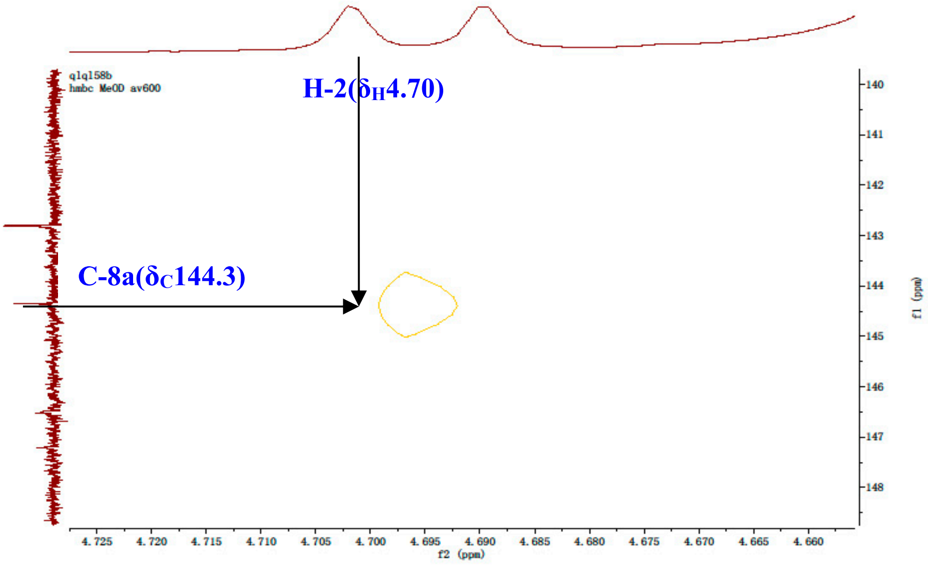

Enlarged HMBC spectrum of **2** (key correlations of H-2/C-8a) in CD_3OD.

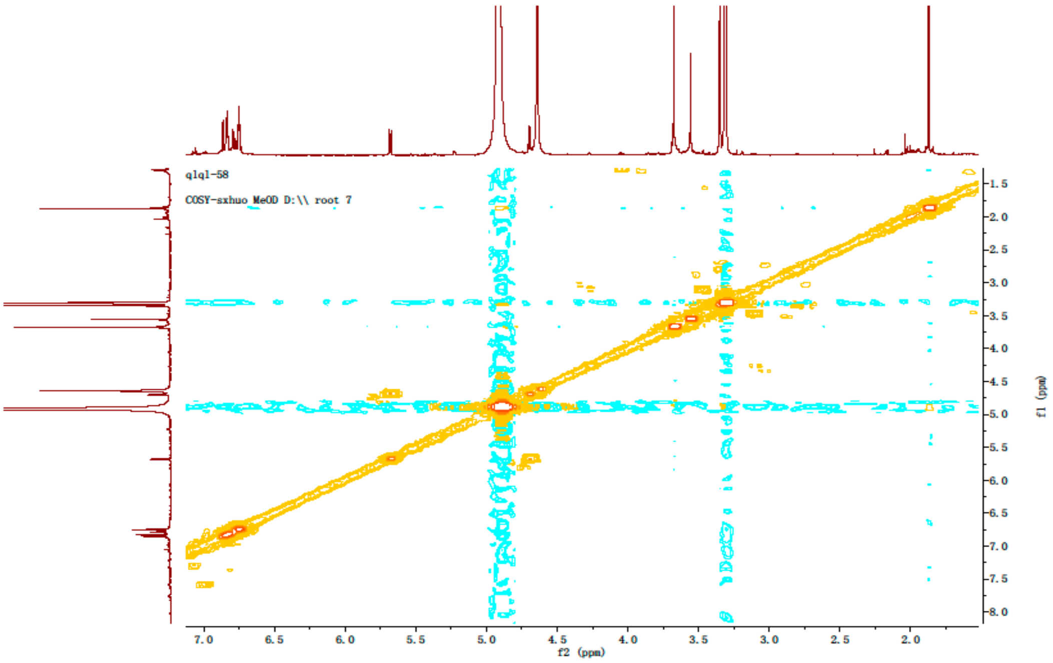

Figure S11. ^{1}H-^{1}H COSY spectrum of **2** in CD_3OD.

Qualitative Analysis Report

**Data Filename**	qlql-58.d	**Sample Name**	qlql-58
**Sample Type**	Sample	**Position**	P1-A3
**Instrument Name**	Instrument 1	**User Name**	
**Acq Method**	SIBU.m	**Acquired Time**	4/13/2015 2:10:14 PM
**IRM Calibration Status**	Success	**DA Method**	Default.m
**Comment**			

Sample Group **Info.**
Acquisition SW 6200 series TOF/6500 series
Version Q-TOF B.05.01 (B5125.2)

User Spectra

Fragmentor Voltage	Collision Energy	Ionization Mode
135	0	ESI

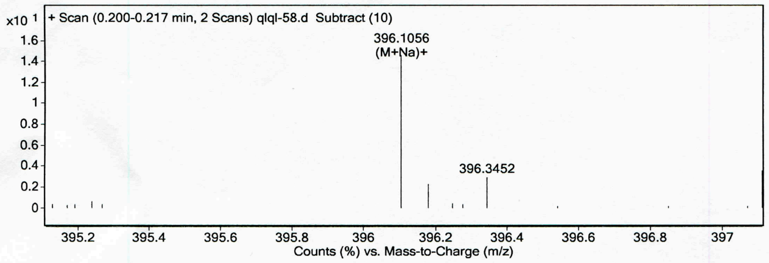

Peak List

m/z	z	Abund	Formula	Ion
274.2744	1	27249.32		
302.3056	1	11985.32		
318.2997	1	14544.99		
330.3365	1	7923.88		
396.1056	1	10285.74	C19 H19 N O7	(M+Na)+
453.1669	1	8345.76		

Formula Calculator Element Limits

Element	Min	Max
C	3	60
H	0	120
N	0	10
O	0	30

Formula Calculator Results

Formula	CalculatedMass	CalculatedMz	Mz	Diff. (mDa)	Diff. (ppm)	DBE
C19 H19 N O7	373.1162	396.1054	396.1056	-0.6	-1.6	11.0000

--- End Of Report ---

Figure S12. HRESIMS spectrum of **2**.

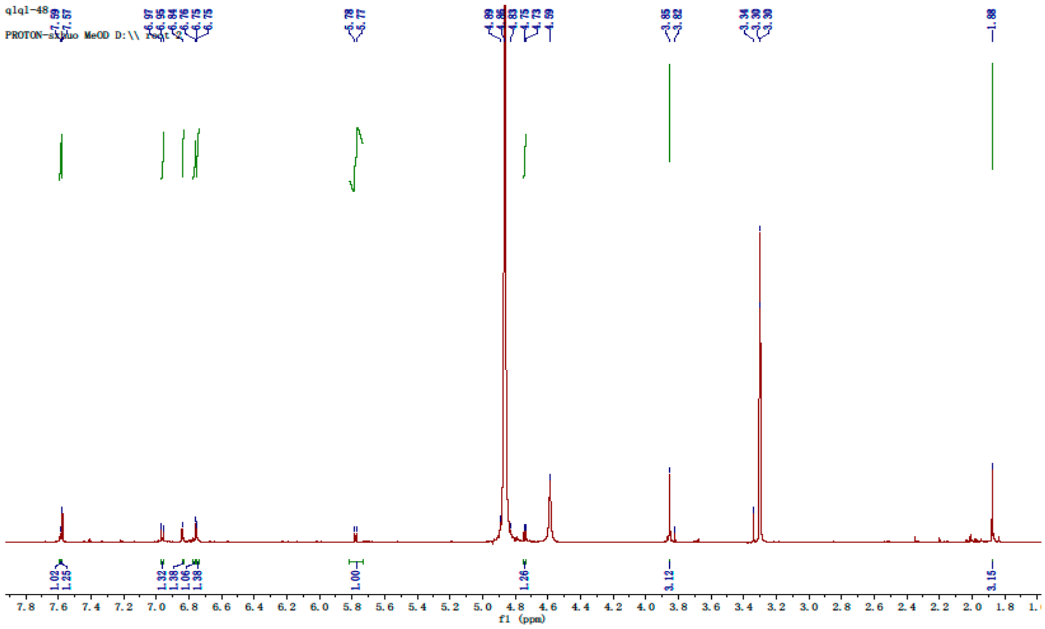

Figure S13. ^{1}H-NMR spectrum of **3** in CD_3OD.

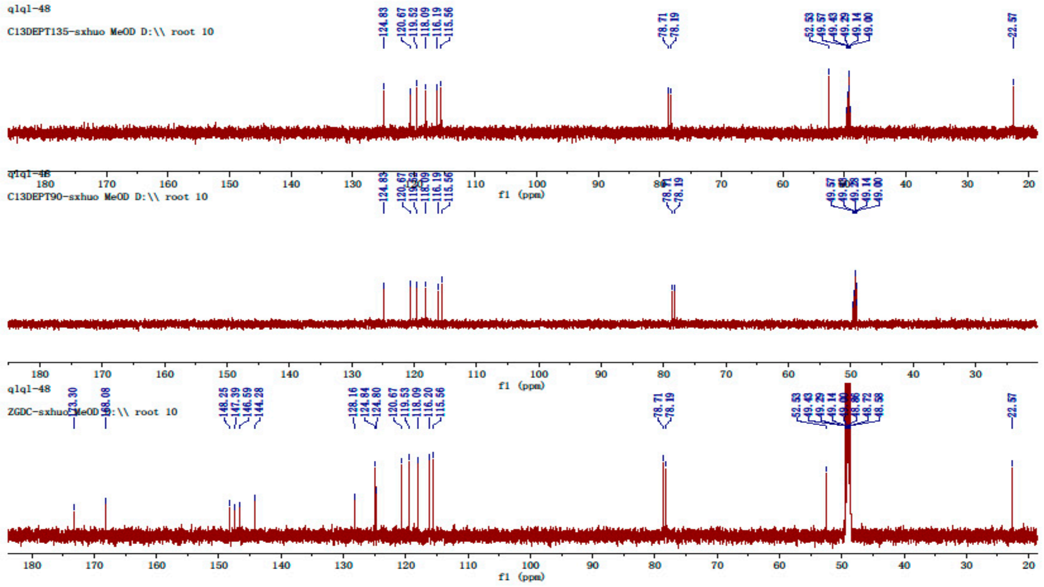

Figure S14. ^{13}C-NMR and DEPT spectra of **3** in CD_3OD.

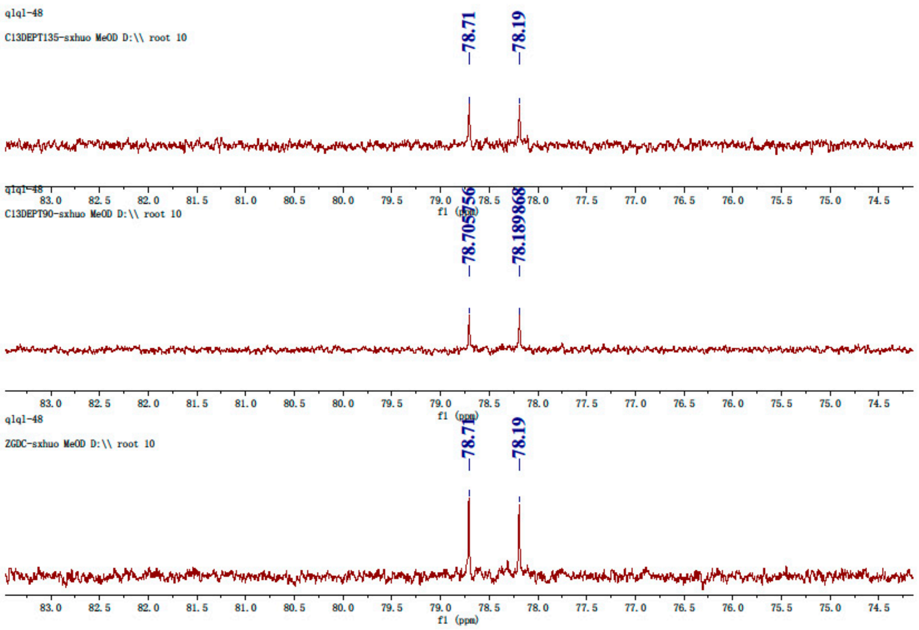

Enlarged ^{13}C-NMR and DEPT spectra of **3** (C-2, C-3) in CD_3OD.

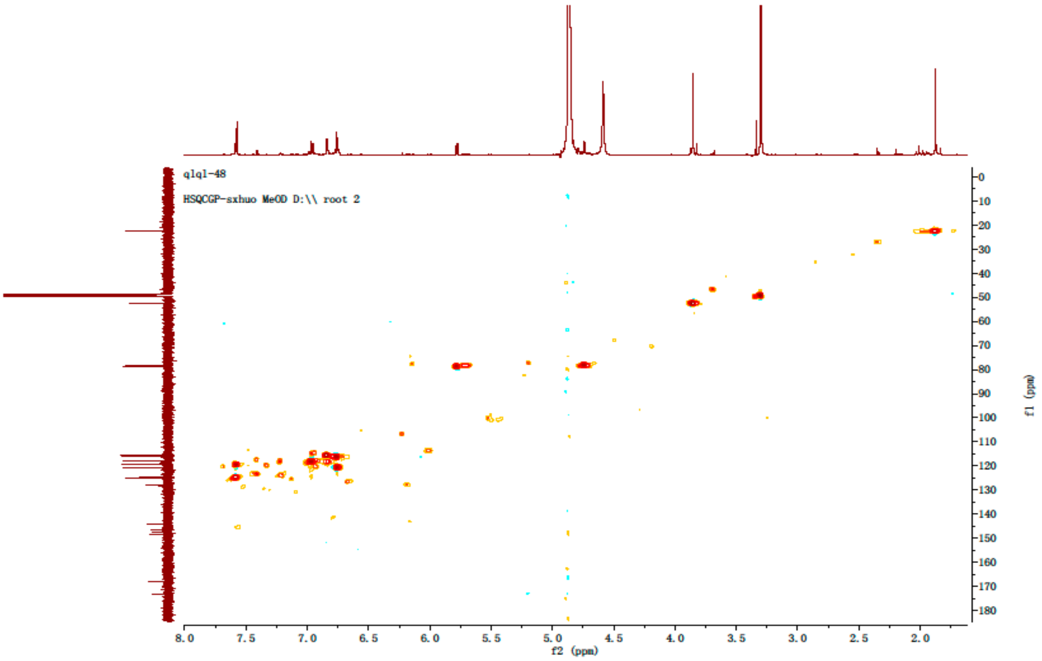

Figure S15. HSQC spectrum of **3** in CD_3OD.

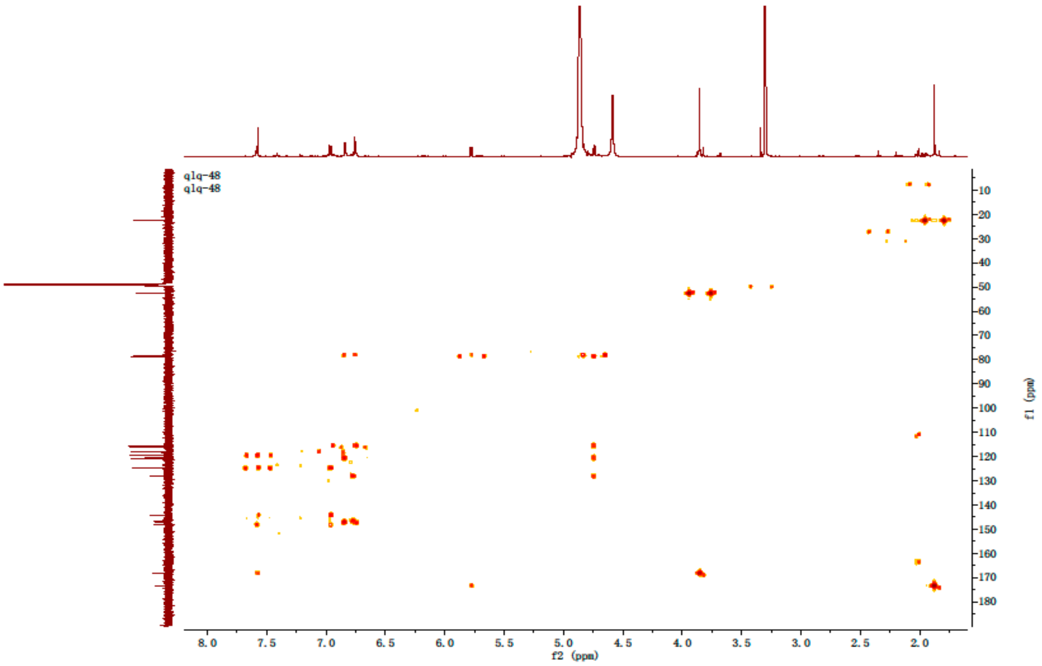

Figure S16. HMBC spectrum of **3** in CD_3OD.

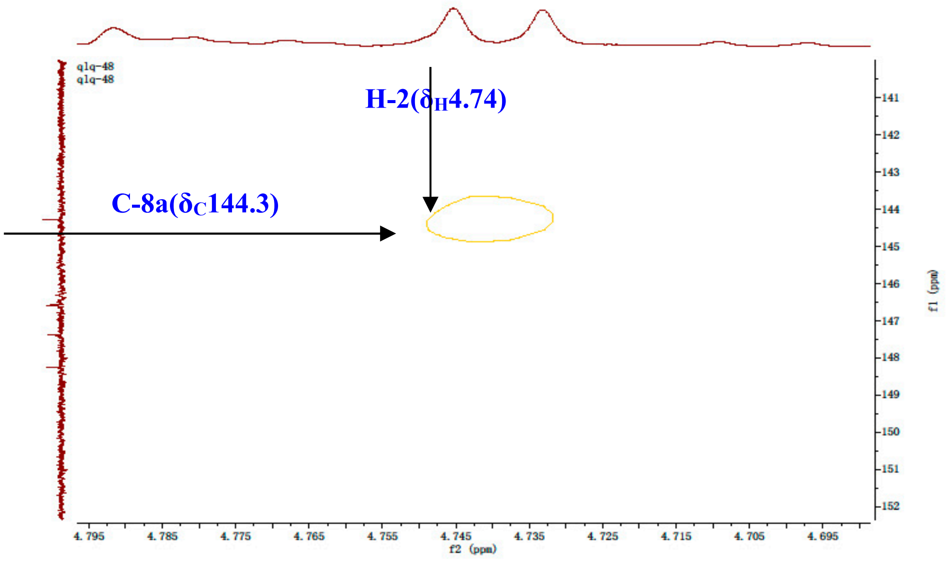

Enlarged HMBC spectrum of **3** (key correlations of H-2/C-8a) in CD_3OD.

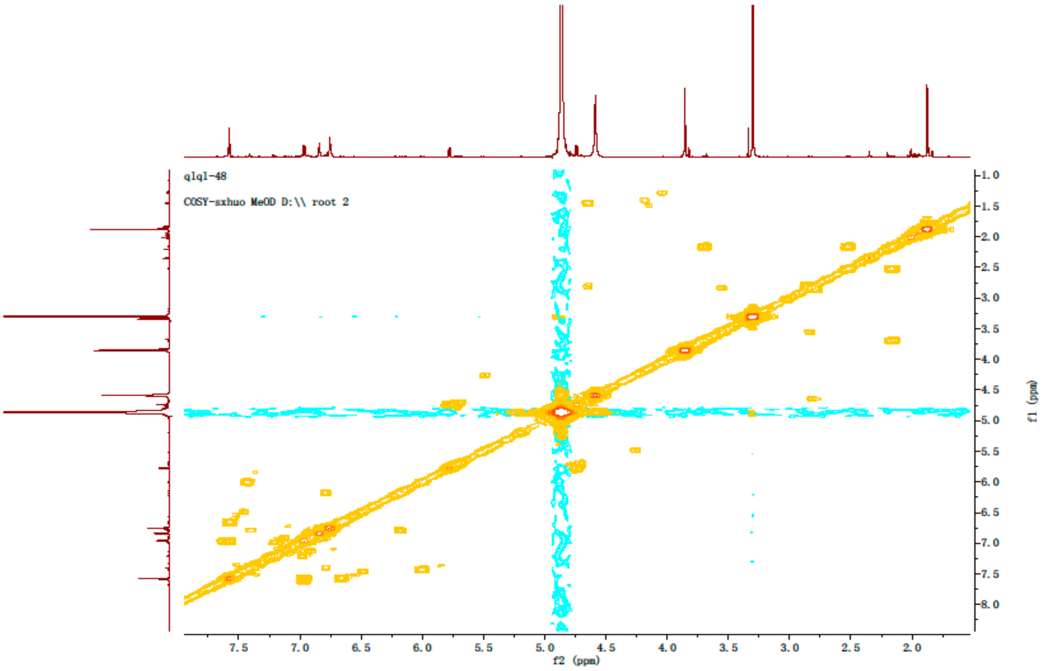

Figure S17. ^{1}H-^{1}H COSY spectrum of **3** in CD3OD.

Qualitative Analysis Report

**Data Filename**	qlql-48.d	**Sample Name**	qlql-48
**Sample Type**	Sample	**Position**	P1-A2
**Instrument Name**	Instrument 1	**User Name**	
**Acq Method**	SIBU.m	**Acquired Time**	4/13/2015 2:08:21 PM
**IRM Calibration Status**	Success	**DA Method**	Default.m
**Comment**			

**Sample Group**		**Info.**
**Acquisition SW**	6200 series TOF/6500 series	
**Version**	Q-TOF B.05.01 (B5125.2)	

User Spectra

Fragmentor Voltage	Collision Energy	Ionization Mode
135	0	ESI

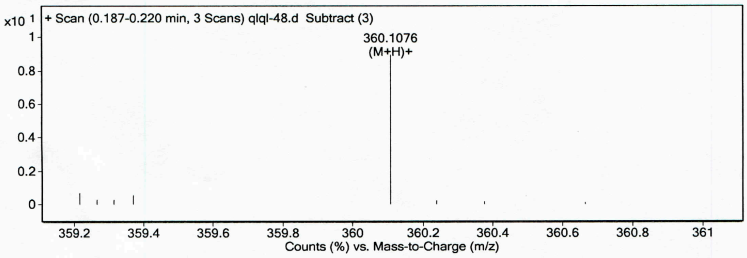

Peak List

m/z	z	Abund
274.2743	1	26317.84
302.3053	1	11388.74
318.3003	1	14657.39
330.3369	1	9079.19
380.2071	1	24642.24
396.1808	1	13134.54

Formula Calculator Element Limits

Element	Min	Max
C	3	60
H	0	120
N	0	10
O	0	30

Formula Calculator Results

Formula	CalculatedMass	CalculatedMz	Mz	Diff. (mDa)	Diff. (ppm)	DBE
C18 H17 N O7	359.1005	360.1078	360.1076	-0.1	-0.3	11.0000

--- End Of Report ---

Figure S18. HRESIMS spectrum of **3**.

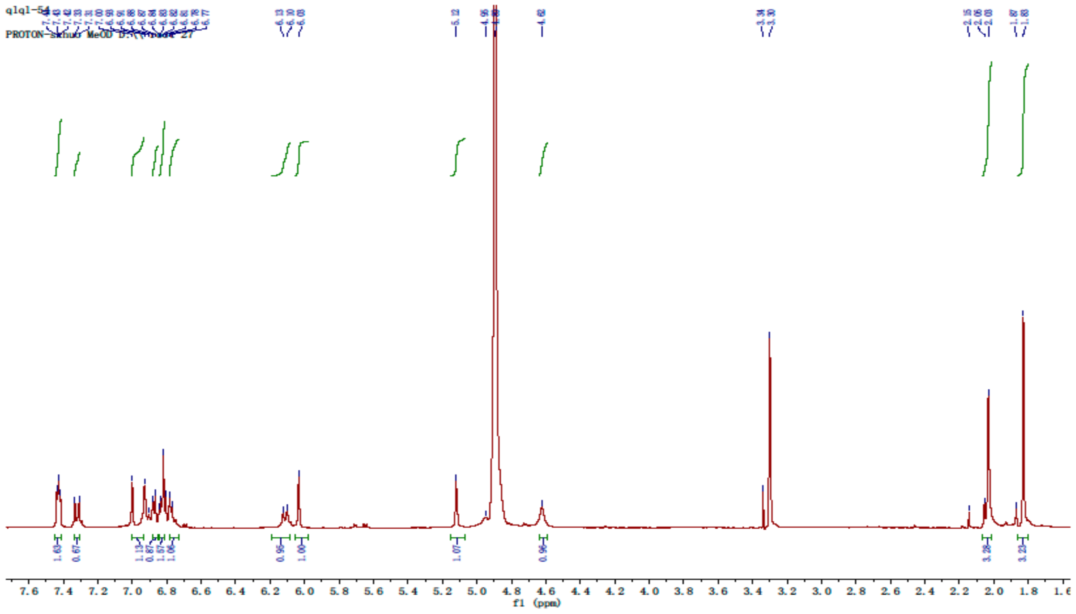

Figure S19. ^{1}H-NMR spectrum of **4** in CD_3OD.

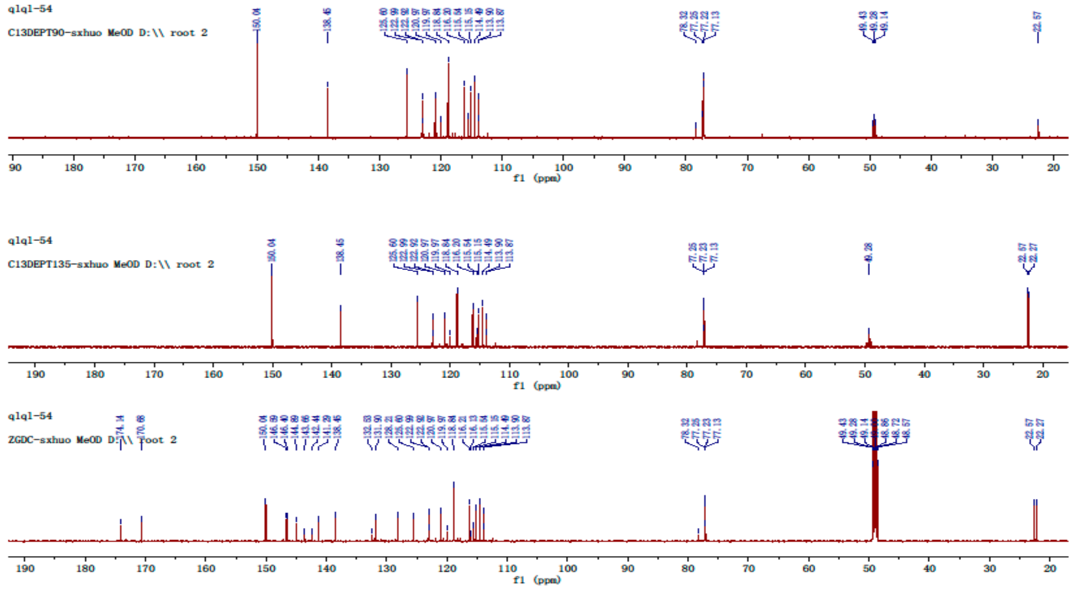

Figure S20. ^{13}C-NMR and DEPT spectra of **4** in CD_3OD.

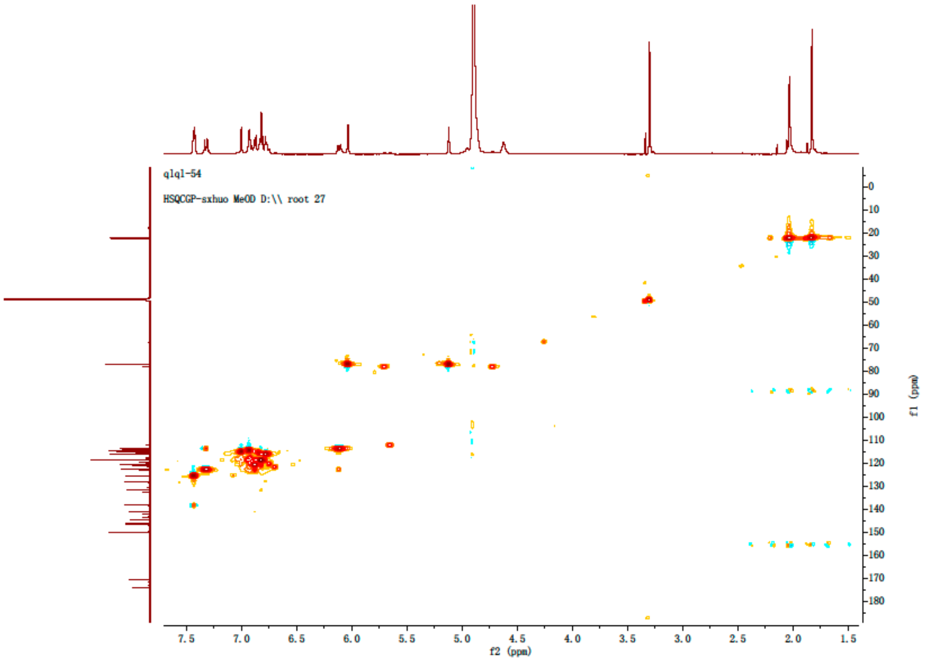

Figure S21. HSQC spectrum of **4** in CD_3OD.

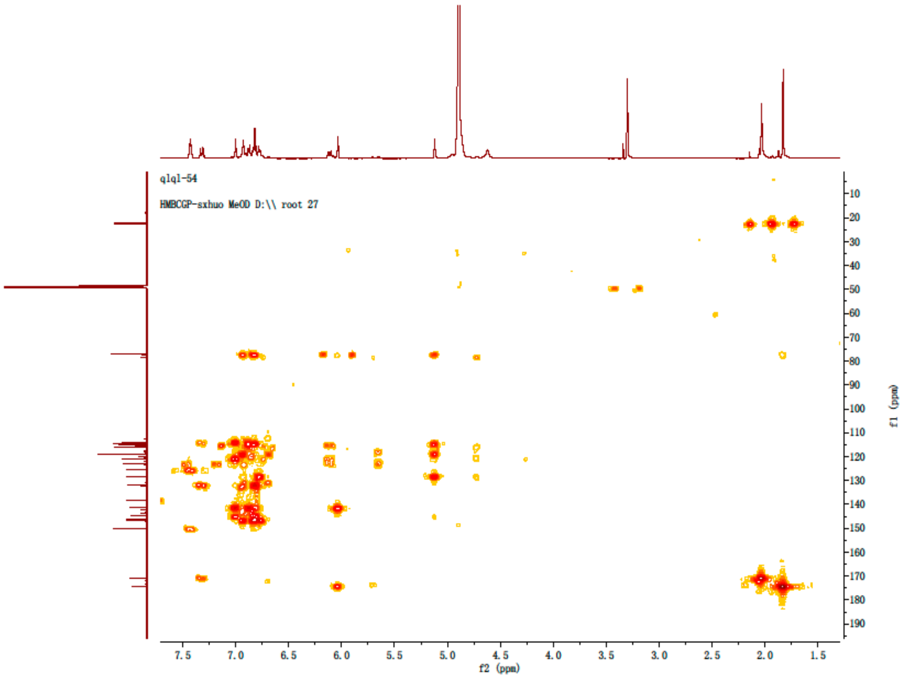

Figure S22. HMBC spectrum of **4** in CD_3OD.

Table S1. The ^{1}H- (600 MHz) and ^{13}C-NMR (150 MHz) spectroscopic data of **4** in CD_3OD.

**Position**	**4**	
	**δ_H (*J* in Hz)**	**δ_C, mult**
2	6.33, d, 7.2	77.1, CH
3	6.03, d, 7.2	77.2, CH
5	6.92, d, 9.0	113.9, CH
6	6.85, dd, 9.0, 1.8	125.6, CH
7		131.9, qC
8	7.00, d, 1.8	118.8, CH
1′		128.2, qC
2′	6.82, d, 2.0	115.1, CH
3′		146.4, qC
4′		146.6, qC
5′	6.81, d, 8.2	116.2, CH
6′	6.76, dd, 8.2, 2.0	120.9, CH
1″	5.12, d, 7.6	113.9, CH_2
2″	7.32, d, 7.6	122.9, CH_2
3″		170.7, qC
4″	2.04, s	22.3, CH_3
3a		174.1, qC
3b	1.84, s	22.6, CH_3
4a		141.3, qC
8a		144.9, qC

Table S2. COX-1 and COX-2 inhibitory activities of the compounds.

**Compd.**	**IC_{50} (μM)**	
	**COX-1**	**COX-2**
**1**	NA [a]	NA
**2**	NA	NA
**3**	NA	NA
**4**	78.85	6.43
**5**	NA	NA

[a] NA: no activity.

Table S3. Cytotoxicity activities of the compounds

**Compd.**	**IC_{50} (μM)**							
	**K562**	**MCF-7**	**A549**	**H1975**	**Hela**	**DU145H**	**Huh-7**	**A431**
**1**	NA [a]	NA	NA	>30	NA	NA	>30	>30
**2**	NA	NA	NA	NA	NA	NA	>30	>30
**3**	NA	NA	NA	NA	NA	NA	>30	NA
**4**	NA	NA	NA	NA	NA	NA	NA	NA
**5**	NA	NA	NA	NA	NA	NA	NA	NA

[a] NA: no activity.

Table S4. MDCK cell-based anti-influenza activities of the compounds.

Compd.	IC_{50} (μM)	
	H1N1	H3N2
**1**	NA [a]	NA
**2**	NA	NA
**3**	NA	NA
**4**	NA	NA
**5**	NA	NA

[a] NA: no activity.

Table S5. EV71 inhibitory activities of the compounds at 30 μM.

Compd.	Inhibition %
**1**	1.7
**2**	1.7
**3**	1.4
**4**	1.2
**5**	2.2
